# Altering SARS Coronavirus Frameshift Efficiency Affects Genomic and Subgenomic RNA Production

**DOI:** 10.3390/v5010279

**Published:** 2013-01-18

**Authors:** Ewan P. Plant, Amy C. Sims, Ralph S. Baric, Jonathan D. Dinman, Deborah R. Taylor

**Affiliations:** 1 Laboratory of Emerging Pathogens, Division of Transfusion-Transmitted Diseases, Food and Drug Administration, Bethesda, Maryland 20892, USA; E-Mail: Ewan.Plant@fda.hhs.gov (E.P.); 2 Departments of Epidemiology, University of North Carolina, Chapel Hill, North Carolina 27599, USA; E-Mails: sims0018@ad.unc.edu (A.S.); rbaric@email.unc.edu (R.B.); 3 Department of Microbiology and Immunology, University of North Carolina, Chapel Hill, North Carolina 27599, USA; 4 Department of Cell Biology and Molecular Genetics, University of Maryland, College Park, Maryland 20742, USA; E-Mail: dinman@umd.edu (J.D.)

**Keywords:** SARS, severe acute respiratory syndrome, pseudoknot, ribosomal frameshifting, viral replication

## Abstract

In previous studies, differences in the amount of genomic and subgenomic RNA produced by coronaviruses with mutations in the programmed ribosomal frameshift signal of ORF1a/b were observed. It was not clear if these differences were due to changes in genomic sequence, the protein sequence or the frequency of frameshifting. Here, viruses with synonymous codon changes are shown to produce different ratios of genomic and subgenomic RNA. These findings demonstrate that the protein sequence is not the primary cause of altered genomic and subgenomic RNA production. The synonymous codon changes affect both the structure of the frameshift signal and frameshifting efficiency. Small differences in frameshifting efficiency result in dramatic differences in genomic RNA production and TCID_50_ suggesting that the frameshifting frequency must stay above a certain threshold for optimal virus production. The data suggest that either the RNA sequence or the ratio of viral proteins resulting from different levels of frameshifting affects viral replication.

## 1. Introduction

*Coronaviridae*, along with *Arteriviridae* and *Roniviridae*, belong to the order Nidovirales. Viruses belonging to these families are large positive strand RNA viruses and are known to infect mammals, birds, fish and arthropods [1]. Entry into a host cell is usually mediated by an interaction between the virus spike glycoprotein and a cellular receptor [2]. After entry, the virus disassembles and a replication/transcription complex forms on double-membraned vesicles ([3] and references within). New subgenomic RNA is produced by a mechanism known as discontinuous transcription [4]. Coronavirus replication requires the production of negative-strand RNA from which positive-strand RNA is produced. Viral proteins are produced from the positive-strand subgenomic RNAs and from the positive-strand full-length RNA. 

The two largest open reading frames, ORF1a and ORF1a/b, are translated from the full-length RNA. These open reading frames (ORFs) encode polyproteins pp1a and pp1ab which are cleaved by self-encoded proteases. The proteins encoded in ORF1a and ORF1a/b function as the replicase, making subgenomic RNAs and new copies of the genomic RNA [5]. Production of the pp1ab polyprotein requires the translating ribosome to change reading frame at the frameshift signal that bridges ORF1a and ORF1a/b. Like most viral frameshift signals, frameshifting at the coronavirus signal leads to expression of an RNA-dependent RNA polymerase (RdRP), a protein essential for viral replication (for review, see [6]). The proteins upstream of the frameshift signal include the predicted proteases and other uncharacterized proteins [5]. We have previously suggested that the ratio of the pp1a and pp1ab proteins might affect the regulation and production of genomic and subgenomic RNA [7].

The SARS coronavirus frameshift signal has a seven nucleotide ‘slippery sequence’ and a stimulatory pseudoknot separated by a spacer region. During programmed -1 ribosomal frameshifting (-1PRF), the tRNAs positioned on the slippery site uncouple from the mRNA and reconnect in the new reading frame. The second stem of the stimulatory pseudoknot is formed by the distal 3’ sequence base-pairing with residues in the loop region of the first stem loop. Unlike other frameshift-stimulating pseudoknots the SARS pseudoknot contains an additional internal stem loop [8,9,10]. The function of this structure, called stem 3, is unknown. We have shown that alterations to the SARS coronavirus frameshift signal affect frameshifting efficiency [9,11]. Reduction in frameshifting efficiency is expected to result in decreased expression of the frameshift proteins, including the RdRP. Some mutations that reduced frameshifting were associated with a several-fold reduction in the amount of genomic RNA [7]. Unfortunately, the mutations employed in prior studies altered both the nucleic acid and protein sequences making it impossible to separate the contributions of RNA and protein. Here, analyses using synonymous protein coding mutations demonstrate that the region of the genome that harbors the frameshift signal affects the regulation of genomic and subgenomic RNA production without altering protein sequence. We discuss possible reasons for this.

## 2. Results and Discussion

### 2.1. Stem 3 Length does not Reduce Frameshifting Efficiency

In an effort to further our understanding of how the SARS frameshift signal functions we performed deletion and mutagenesis studies an analyzed the effects on frameshifting efficiency, RNA structure and viral RNA production. There are at least three different types of structures used by the coronaviruses to stimulate frameshifting. Some coronaviruses, such as SARS-CoV, use a three stemmed pseudoknot, others such as human coronavirus 229E have an ‘elaborated pseudoknot’ or kissing stem-loops, while the avian infectious bronchitis virus utilizes a two stemmed pseudoknot [9,12,13]. That these diverse coronaviruses retain some sequence between stems 1 and 2 yet have quite different frameshift stimulating structures is intriguing. One inference that can be made is that the RNA sequence, or a structure formed by it, is involved in some other aspect of the virus lifecycle. Our initial hypothesis was that a regulatory element was contained within this sequence. Here we describe deletion and mutagenesis experiments with a dual luciferase reporter to show that the effect the sequence between stems 1 and 2 has on frameshifting efficiency is due to structural changes those mutations cause in the pseudoknot. Further, we show by making synonomous mutations in the virus that different levels of frameshifting efficiency affect the production of genomic and subgenomic RNA differently. This could be caused by disruption of a regulatory element or the altered ratio of proteins derived from the coding region flanking the frameshift signal.

The individual residues and each stem in the SARS pseudoknot make unique contributions to frameshifting efficiency [8,9,10]. For example, while stem 2 is essential for frameshifting, stem 3 is not. Even small changes in Stem 2 (e.g., replacing or deleting the bulged adenosine in stem 2 with a cytosine) reduced frameshifting to levels similar to those observed with the complete disruption of stem 2 [9]. In contrast, altering the bulged adenosine in stem 3 or disruption of stem 3 promoted only modest changes in frameshifting [9]. These observations engendered the hypothesis that stem 3 formation may affect frameshifting by enhancing or facilitating the formation of stem 2. 

**Figure 1:**
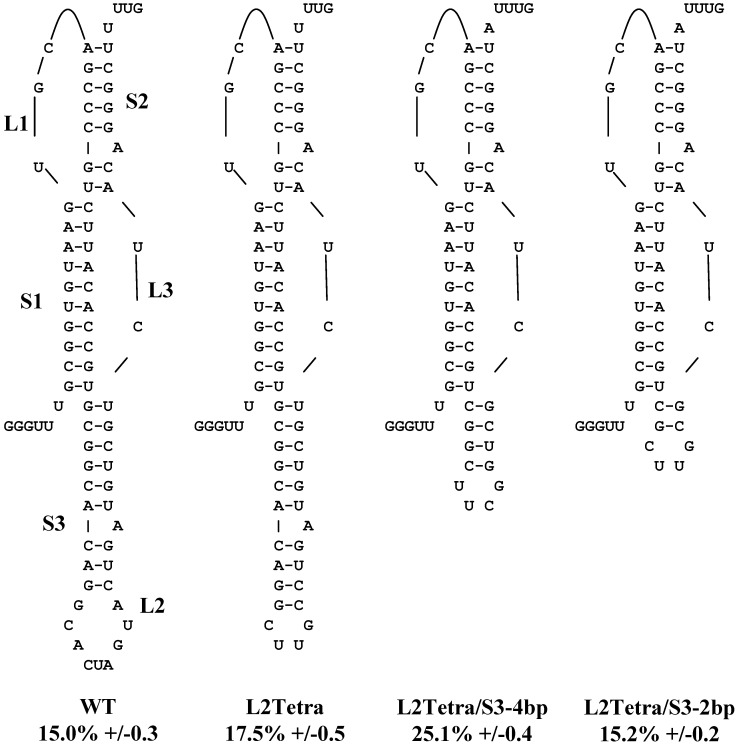
Removing stem 3 does not reduce programmed -1 ribosomal frameshifting (-1 PRF) efficiency. The predicted secondary structure of the SARS coronavirus pseudoknot is shown, along with a series of mutants with stem 3 truncated. The stems are labeled S1 to S3 and the loops labeled L1 to L3. Site directed mutagenesis was used to replace the wild-type loop 2 with a CUUG tetraloop (L2Tetra). Additional truncations to stem3 were made (L2Tetra/S3-4bp and L2Tetra/S3-2bp) and frameshifting efficiency analyzed by dual luciferase assay. Frameshifting efficiency is expressed as a percentage with standard error as described in the experimental section.

Experiments were designed to test the effects of changes to the length of stem 3 while still maintaining a stable structure. Beginning with the wild-type stem 3 containing nine paired bases and a nine residue loop, the following series of mutants were constructed. First, the loop capping stem 3 was replaced with a shorter tetraloop; the L2Tetra construct contains ten paired bases and a four residue loop. This was used as the basis for construction of two additional tetraloop constructs with progressively shorter stem 3 structures: L2Tetra/S3-4bp, containing only four paired bases in the stem, and L2Tetra/S3-2bp, which has only two paired bases. The 5’-uucg-3’ tetraloop sequence used in the L2Tetra/S3-4bp mutant was selected because the last base-pair before the tetraloop affects its formation. The closing pair for UNCG tetraloops is usually C-G compared to the G-C closing base-pair found in CUUG tetraloops. All of the truncated structures promoted frameshifting at levels equal to or greater than the wild-type pseudoknot structure using a dual luciferase reporter assay (Figure 1). Interestingly, the L2Tetra/S3-4bp mutant stimulated frameshifting at 25.1% compared to the 15 to 17.5% observed for the other stem 3 mutants. It is possible that the difference in the tetraloop contributed to the slight increase in frameshifting efficiency. These results, and experiments reported by Brierley *et al.*, for another coronavirus [12] indicate that the length of stem 3 does not play a critical role in frameshifting, suggesting that it may serve some other function. 

### 2.2. Loop 2 is Important for Efficient Frameshifting

**Figure 2:**
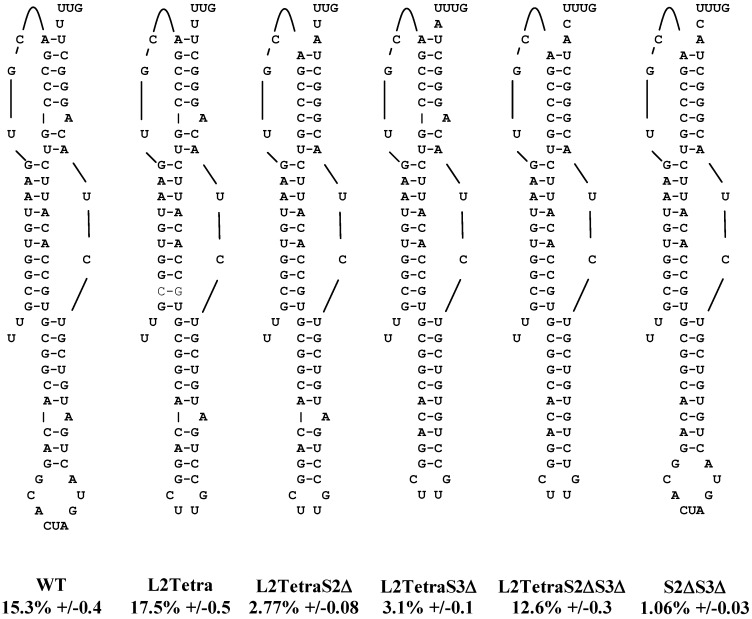
Stabilizing stem 3 with a tetraloop preserves -1 PRF efficiency. Site directed mutagenesis was used to change loop2 of the wild-type SARS pseudoknot into tetraloop (L2Tetra from Figure 1). The bulged adenosines in stems 2 and 3 were removed by site directed mutagenesis singly or together in conjunction with the tetraloop (L2TetraS2Δ, L2TetraS3Δ, and L2TetraS2ΔS3Δ). Both bulged adenosines were removed from the wild-type pseudoknot (S2ΔS3Δ). Frameshifting efficiency is expressed as a percentage with standard error as described in the experimental section.

While alteration of the bulged adenosine in Stem 2, or disruption of Stem 2 primary and secondary structures dramatically reduces frameshifting efficiency, neither the bulged adenosine in Stem 3, nor the sequence of Stem 3 itself, have similarly strong roles [9,11]. However, given the ability of tetraloop-capped stem 3 mutants to promote high levels of frameshifting, we asked if these mutations could compensate for deletion of the bulged adenosine in stem 2. To this end, the bulged adenosines in stems 2 and 3 were deleted both individually and together in the context of the L2Tetra construct (Figure 2). L2Tetra was chosen as the baseline because, among the tetraloop mutants, it most closely represents the wild-type sequence. Deletion of the bulged adenosine in stem 2 (L2TetraS2Δ) reduced frameshifting by 84.2% (17.5% *versus* 2.77%, *p* = 9.3 × 10^−19^, Figure 2). When the bulged adenosine from stem 3 was removed (L2TetraS3Δ) a similar reduction of 82.5% was observed (17.5% *versus* 3.1%, *p* = 1.1 × 10^−19^). Interestingly, removal of both adenosines (L2TetraS2ΔS3Δ) partially restored frameshifting with only a 28.2% reduction in frameshifting (17.5% *versus* 12.6%, *p* = 7.5 × 10^−5^). Bulged adenosines, which are known to bend helices [14], may be participating in formation of a functional pseudoknot structure. Indeed, many frameshift-stimulating structures have bent conformations that are postulated to help effect frameshifting [15,16,17]. Importantly, however, the contribution of the bulges in the SARS pseudoknot is not the same in the context of the native stem 3/loop 2. Specifically, removing both bulged adenosines (S2ΔS3Δ) from the wild-type backbone decreased rates of frameshifting by 93% (15.3 *versus* 1.06%, *p* = 1.7 × 10^−18^), similar to that observed with the removal of the stem 2 bulge alone (Figure 2). Together these results suggest that stabilization of stem 3 assists in the formation or stability of stem 2, which in turn is essential for frameshifting. However, the actual sequence of loop 2 or the tetraloop-capping stem 3 also affected frameshifting efficiency, either through the stabilization of stem 3 or the formation of stem 2 itself, thus implicating the nucleotides in loop 2 or the tetraloop in tertiary structure interactions. Further evidence comes from the chemical protection data (below), demonstrating that the altered sequence of loop 2 affects the structure of stems 2 and 3. Although secondary structure predictions indicate that the nucleotides in loop 2 are not part of a helix, those nucleotides are susceptible to both single- and double-strand-specific nucleases [9] suggesting that these nucleotides participate, at least part of the time, in some ordered structural state. 

### 2.3. Destabilizing Stem 3 Base-Pairing Adversely Affects Frameshifting

While the high degree of phylogenetic conservation of stem 3 in many coronaviruses [9] suggests that it serves an important function, mutations in this element have less impact on frameshifting efficiency than mutations in stem 2 [9,11,12]. Prior structural analyses also suggested that the stability of the structure may contribute to frameshifting efficiency. Here, specific changes were made to Stem 3 in order to directly address this issue. 

**Figure 3:**
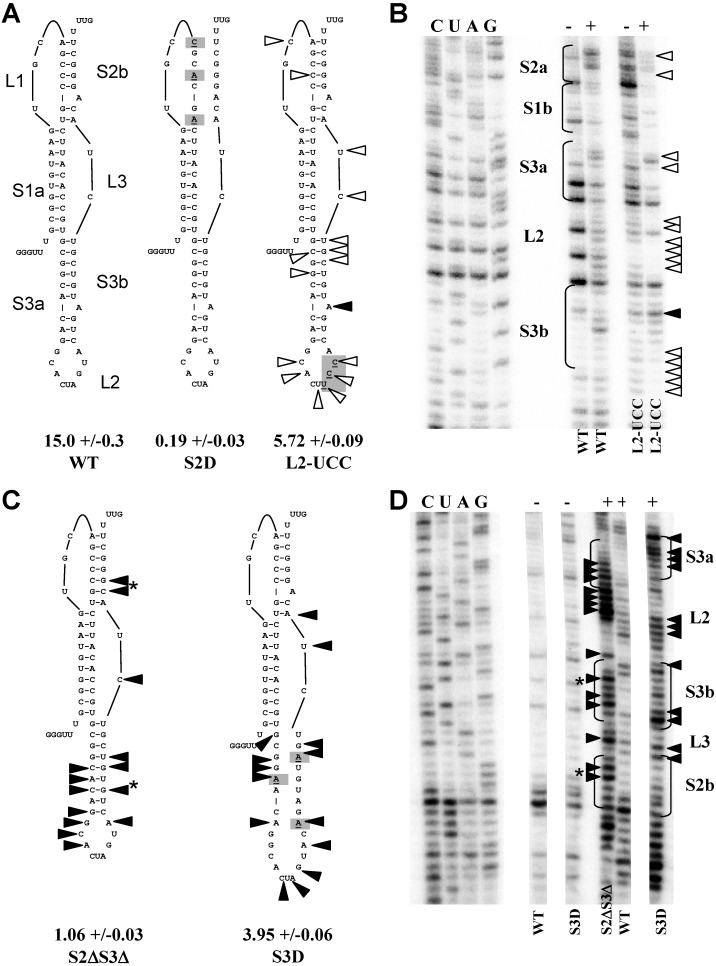
Mutations to stem 3 alter pseudoknot structure. (**A**) The sequence and structure of the wild-type SARS coronavirus pseudoknot is shown on the left (WT). The stems are labeled S1 to S3 with the 5’ portion denoted ‘a’ and the 3’ portion ‘b’. The loops connecting the stems are labeled L1 and L3, and the loop capping the third stem is labeled L2. Changes made to the pseudoknot are shaded and underlined in mutants S2D and L2-UCC. The efficiency of frameshifting as determined by dual luciferase assay is expressed as a percentage below each mutant with standard error shown. (**B**) Comparison of L2-UCC and WT pseudoknots by SHAPE analysis (see Experimental Section). An autoradiograph of the primer extension for the wild-type and L2-UCC pseudoknots is shown. The sequencing ladder is shown on the left side of the gel and SHAPE reactions are shown on the right. Reactions performed with (+) or without (-) NMIA are indicated. The closed carrots show increased reactivity and the open carrots show reduced reactivity. (**C**) Diagrams of pseudoknots with mutations (shaded and underlined) in Stem 3. Asterisks mark the position of deleted adenosines. Frameshifting efficiency is shown below each mutant. Positions of increased or decreased NMIA reactivity are indicated. (**D**) SHAPE analysis of stem 3 mutants. The position of the stems is indicated on the gel, note that because S2ΔS3Δ contains deletions the position of the stems differ slightly from the S3D stems. The changes for S2ΔS3Δ are indicated on the left and the changes to S3D are indicated on the right of the gel.

A negative control plasmid was constructed in which Stem 2 base-pairing was disrupted (S2D) by mutating three residues in the third codon position so as to retain the amino acid sequence of the frameshift protein pp1ab (Figure 3A). Consistent with prior studies [9,11,18], disruption of stem 2 resulted in a 99% reduction in frameshifting (15% *versus* 0.19%, Figure 3A) demonstrating that the integrity of the second stem, and hence the pseudoknot, is required for efficient frameshifting. A control plasmid, L2-UCC, with a synonymous mutation in loop 2 was also made. The AGU codon was changed to UCC because the latter is present at the same position in the TGEV coronavirus and would be expected to have a minimal impact. More detailed structural information about the L2-UCC mutant is published elsewhere [19]. As expected, a moderate change in frameshifting efficiency was observed (5.72% *versus* 15%, Figure 3A).

Next, a stem 3 mutant was designed to alter the structure of stem 3 yet retain the same coding sequence so that the effects of these mutations on virus propagation could also be tested *in vivo* (Figure 3C). In the S3D construct, Stem-3 base-pairing was disrupted. The observed effects *in vivo* should therefore reflect changes related to RNA structure within the frameshifting signal, rather than changes in the function of the encoded proteins. The silent codon changes in S3D promoted moderate reductions in -1 PRF efficiency as compared to the silent S2 mutants (3.95% *versus* 0.19%; compare Figure 3A and 3C. These moderate changes are similar to the L2-UCC mutant and those observed for other stem 3 mutants [9,11]. Importantly these values are higher than 2.33%, the lowest level of frameshifting previously observed for a viable SARS coronavirus [7]. 

### 2.4. Destabilizing Stem 3 Base-Pairing Alters the Pseudoknot Structure

To determine if the synonymous mutations altered the structure of the pseudoknot, their effects on this structure were evaluated using selective 2’-hydroxyl acylation analyzed by primer extension (SHAPE) analysis (see Experimental Section). The negative control S2D did not form apseudoknot-like structure (data not shown). The L2-UCC loop is more structured than the wild-type loop and this reduced flexibility appeared to affect the remaining structure of the pseudoknot as shown by diminished reactivity to NMIA at and around the mutated bases in the loop (L2) compared to the wild-type structure (Figure 3B). Additionally, there was decreased reactivity with some bases in stem 3 and in the loops L1 and L3. These data correlate well with prior chemical analysis showing that the nucleotides in Loop 2 were susceptible to both single- and double-strand-specific nucleases [9].

Having established this baseline, S3D and S2ΔS3Δ mutants were evaluated by SHAPE. In contrast to the L2-UUC pseudoknot mutant, the S3D and S2ΔS3Δ pseudoknots were more reactive to NMIA indicating that they are less structured (Figure 3D). In addition to the expected changes in reactivity to the mutated bases in stem 3, bases in the 3’ region of both loop 2 which caps stem 3 and the loop joining stems 2 and 3 (L3) also showed increased reactivity for S3D. The S2ΔS3Δ mutant was also more reactive to NMIA. However, the NMIA reactivity pattern of the S3D mutant, with more reactivity in the 5’ proximal end of stem 3 and the 3’ portion of loop 2, differed from that seen for S2ΔS3Δ in which reactivity was more clustered in the 5’ portion of loop 2 and adjacent stem 3 nucleotides. These data suggest that the native structure of loop 2 and correct formation of stem 3 is dependent on the presence of the bulged adenosines. 

### 2.5. Destabilizing Stem 3 Base-Pairing Alters Genomic and Subgenomic RNA Production

**Figure 4:**
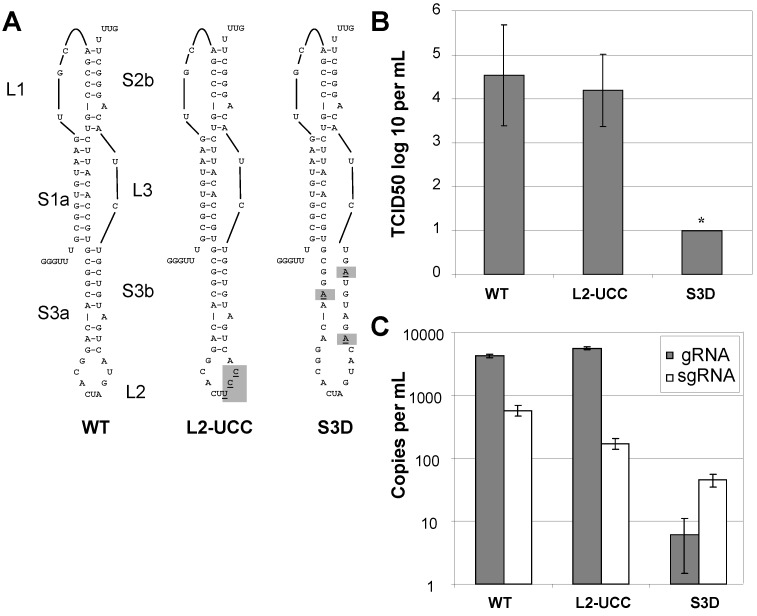
Mutations to stem 3 can affect virus infectivity and replication. (**A**) Schematic of the wild-type SARS pseudoknot and viral mutants. The specific bases that were altered are shaded and underlined. (**B**) Tissue culture infectious dose was determined as described in the materials and methods. The values shown are the average of six measurements with error bars showing the standard deviation. Asterisks indicate values that were at the lower limit of the assay. (**C**) Relative abundance of genomic and subgenomic RNA in viral stocks. Plaque purified virus was used to infect Vero cells. Four days post infection CPE was observed. Media and detached cells were removed. RNA was extracted from a 100μL aliquot using Trizol. Taqman analysis was used to determine the total number of genomic and subgenomic RNA molecules compared to a reference RNA transcribed from a SARS replicon. The number of copies per mL of viral stock is shown with standard deviation.

Although most mutations in stem 3 have a limited impact on frameshifting there is evidence of sequence conservation between coronavirus sequences through this region [9]. To determine the biological significance of stem 3, we tested a virus containing synonymous stem 3 mutations and a virus containing synonymous loop 2 mutations (Figure 4A). RNA was extracted from the infected cell lysate, amplified by RT-PCR and sequenced to confirm that the desired mutations were present. The S3D virus was viable but replicates to a lower titer than the wild type (Figure 4B; Table 1). The effect on production of genomic and subgenomic RNA was quantified by RT-PCR (Figure 4C). Although the S3D virus could be detected by RT-PCR, the TCID_50_ values were below the limit of the assay (Table 1; Figure 4B). The L2-UCC mutant was detected by both methods.

The RT-PCR analysis demonstrates that the genomic RNA (gRNA) of L2-UCC accumulates to wild-type levels four days post-infection (Figure 4C). In contrast, genomic RNA from the S3D mutant only accumulated to levels approximately three orders of magnitude lower. Interestingly, the genomic to subgenomic RNA ratios were inverted for the S3D mutant: while the wild type and L2-UCC viruses had tenfold more genomic than subgenomic RNA, the S3D mutant had tenfold more subgenomic RNA than genomic RNA (Figure 4C). Similar changes in viral RNA ratios were seen with viruses with mutated slippery sites, implicating a broader region of this part of the genome as being involved in regulation of transcription [7]. While the changes in the previous report resulted in an altered protein sequence, it is unknown if the pseudoknot structure in the slippery site mutants was affected. The viruses used in this study all encode identical proteins indicating that protein function itself is not causing the altered transcription patterns. This suggests that the differences between the viruses are due to RNA sequence and/or structural changes.

Because the RT-PCR quantification was performed on the supernatant from cells exhibiting CPE and expected to contain representative amounts of cellular gRNA and sgRNA, it is possible that the RT-PCR data could be indicative of differing amounts of gRNA packaged in virions. The reduction in virion-associated gRNA levels could occur if a packaging signal was disrupted. Disruption of a packaging signal would result in fewer complete virions and lower TCID_50_ and gRNA values as observed for the S3D mutant. Although the efficiency of frameshifting is similar for the L2-UCC and S3D mutants the gRNA levels differ by several log. The stabilization of L2-UCC stem 3 structure and loss of S3D stem 3 structure (see Figure 3) also support the idea that this region may contain a packaging signal.

**Table 1:** Frameshifting efficiency affects infectivity. SARS coronavirus mutants are indicated. -1 PRF efficiencies and standard error as measured in VeroE6 cells as described in the Experimental Section. The data for the WT and L2-UCC viruses has previously been published [7,19]. TCID_50_ values were calculated as described in the Experimental Section. The error is the standard deviation of six measurements.

Mutant	% -1 PRF	TCID_50_
WT	15.0 +/- 0.3	4.5 +/- 1.1
L2-UCC	5.72 +/- 0.09	4.2 +/- 0.8
S3D	3.95 +/- 0.06	< 1.0

## 3. Experimental Section

Construction of dual luciferase plasmids. The parental plasmids pJD464 and pJD502, containing the *Renilla* and firefly luciferase genes flanking the wild-type SARS-CoV frameshift signal, have been described previously [9]. pJD502 is the test construct for measuring frameshifting efficiency, and pJD464 is a readthrough control plasmid to normalize against any defects in overall translation that the introduced SARS sequence may cause. Site-directed mutagenesis was used to introduce mutations into the test construct (pJD502). Mutagenesis was performed using Stratagene’s QuikChange II kit (La Jolla, CA). The mutations were confirmed by sequence analyses.

Construction of mutant viruses. Oligonucleotide site directed mutagenesis was used to mutate SARS subclone D [20]. Each mutant subclone was assembled with the other five subclones, and then T7 RNA polymerase and a GTP cap analog were used to generate a full-length virus transcript as described previously [20]. Full-length genomic RNA was transfected into Vero cells resulting in a productive, lytic viral infection. The virus was allowed to grow for 5 days at 37 °C. Viral supernatants were plated onto Vero cells, and several clones were plaque purified. The plaque-purified viruses were expanded by growth on Vero cells. RT-PCR was used to detect subgenomic RNA, a marker of viral replication. PCR amplicons from the frameshift region were sequenced to verify that the correct mutations were introduced. Virus titer was measured using a TCID_50_ assay calculated according to the method of Reed and Muench [21]. All viral assays were conducted in BSL-3 safety facilities.

The abundance of gRNA and sgRNA was determined by quantitative real-time PCR using SYBR green technology as previously described [7]. Primers complementary to gRNA and sgRNA were used to detect RNA. Although the primers complementary to the sgRNA can anneal to the gRNA, only the smallest sgRNA has the 5’ leader and 3’ sequence close enough to allow PCR amplification under normal cycling conditions.

RNA Structure Probing. The structures of the pseudoknots were probed using the selective 2’-hydroxyl acylation analyzed by primer extension (SHAPE) procedure of Wilkinson *et al.* [22]. SHAPE analysis utilizes N-methylisatoic anhydride (NMIA), which tests the local backbone flexibility of RNA. More flexible nucleotides assume conformations that are reactive to NMIA which irreversibly acylates the ribose 2’-hydroxyl groups. This then presents as a stop upstream of the modified base during primer extension. Thus, a more intense band in the primer extension autoradiograph represents a more flexible region of the RNA. Briefly, DNA was amplified from each mutant plasmid using PCR master mix (Fermentas Inc., Glen Burnie, MD) and a forward-direction primer containing the T7 RNA promoter; T7forSHAPE, 5'-TAATACGACTCACTATAGGGAAGATGCACCTGATGAAATGG-3' and the reverse-direction primer; revSHAPE, 5'-GCCCATATCGTTTCATAGCTTC-3'. RNA was synthesized from the PCR products using the Ambion T7 MEGAscript kit (Austin, TX) as per the manufacturer’s instructions. RNA (2 pmol) in a volume of 12 μL was denatured at 95 ºC and cooled on ice. Reaction buffer (6 μL) containing 333 mM HEPES (4-(2-hydroxyethyl)-1-piperazineethanesulfonic acid) pH 8.0, 20 mM MgCl2, and 222 mM NaCl was added to each RNA sample and the mixture was incubated at 37 ºC for 20 minutes. The reactions were split into two equal aliquots, one with 1 μL Dimethyl Sulfoxide, the other with 1 μL of 65 mM N-methylisatoic anhydride (NMIA), and incubated at 37 ºC for 45 minutes. The RNA was then precipitated with 90 μL H_2_O, 4 μL 5M NaCl, 1 μL 20 mg/mL glycogen, 2 μL 100 mM EDTA pH 8.0, and 350 μL ethanol overnight at −80ºC. After centrifugation the RNA was resuspended in 14 μL of 0.5 × TE. The oligonucleotide 5'-GCCGGGCCTTTCTTTATG-3' (50 pmol; Integrated DNA Technology, Coralville, IA) was labeled with 30 μCi gamma 32 P-adenosine triphosphate (ATP) using T4 kinase (Roche, Indianopolis, IN) and purified through a G-25 column (GE Healthcare). RNA (7 μL) was incubated with 32P-labeled oligonucleotide (3 μL) at 65 ºC for 5 minutes, 35 °C for 5 minutes and cooled to 0 ºC. Reverse transcription reactions were performed using SuperScript III enzyme (Invitrogen, Carlsbad, CA) at 52 ºC for 20 minutes. Products were separated on an 8% polyacrylamide, 7.5M urea gel in TBE buffer, dried and visualized by PhosphorImage analysis.

Dual Luciferase Assays. For each assay cells were transfected with either a test plasmid or a control plasmid (described above) using FuGene6 from Promega (Madison, WI). VeroE6 cells were grown overnight in Dulbecco's Modified Eagle Medium supplemented with 10% FBS at 37 ºC. Cells were disrupted using the passive lysis buffer (Promega, Madison, WI) as per the manufacturer’s instructions. Luminescence reactions were initiated by addition of 10 – 20 μL of cell lysates to 100 μL of the Promega LAR II buffer and completed by addition of 100 μL Stop’n’Glo reagent. Luminescence was measured using a Turner Design TD20/20 instrument. At least three replicates were performed within each assay and all assays were repeated at least three times until the data were normally distributed to enable statistical analyses both within and between experiments. The frequency of frameshifting is expressed as a ratio of firefly to Renilla luciferase from a test plasmid divided by the analogous ratio from the read-through control plasmid multiplied by 100%. Fold change, standard error and estimates of the P-values for ratiometric analyses were performed as described previously [23].

## 4. Conclusions

In line with previous studies of coronavirus frameshift signals, data presented here indicate that the sequence and length of the third stem is not critical for frameshifting efficiency [9,12,18]. However, as revealed by the structural analysis, mutations in stem 3 can cause changes in frameshift efficiency by changing stem 2 formation. Additionally, changes in stem 3 can be detrimental to viral viability. Specifically, changes that destabilize stem 3 resulted in reductions in -1 PRF and altered patterns of viral RNA production. It remains to be determined if the latter change is due to a disruption in frameshifting or disruption of a RNA regulatory element such as a packaging signal.

While many sequences involved in coronavirus subgenomic RNA synthesis have been elucidated, sequences and structures involved in regulating coronavirus genomic RNA synthesis are less well defined [24]. Internal replication elements have been described in other plus-strand non-segmented viruses including Poliovirus, Hepatitis C Virus and Tombusviridae [25,26,27]. Each of these internal replication elements is present in the region of the viral genome encoding non-structural proteins. The Tombusviridae RNA-dependent RNA polymerase is translated as a readthrough product and the internal replication element is downstream of the readthrough signal [25]. Although the CRE element from poliovirus has been studied for several years the precise roles of these elements are not well defined [28]. In most instances it is not known, for example, if they bind to specific viral or host proteins, or if they communicate with other elements in the genome.

Packaging signals for coronaviruses are also ambiguous and have been identified in the 5’ untranslated regions and the 3’ end of ORF1a/b [29,30]. Our work demonstrated that a less structured stem 3 in the frameshift pseudoknot led to significantly reduced amounts of gRNA when compared to viruses with more structured pseudoknots. However, the same change in viral RNA patterns was observed with a slippery site mutant suggesting that if a packaging signal is present it extends from, or is affected by, the upstream slippery site.

The correlation between stem 3 structure and gRNA levels indicates that a structural requirement in stem 3 is required for optimal RNA production but we cannot, at this stage rule out a frameshifting threshold similar to that observed for the yeast double-stranded RNA virus M1 [31]. That is, if frameshifting drops below a certain frequency, the change in the ratio of pp1a to pp1ab results in a dramatic reduction in gRNA production. Indeed, recent reports describing the function of a nonstructural protein (nsp8) encoded upstream of the frameshift signal provide an alternative hypothesis to regulatory element hypothesis investigated in this work. Nsp8 was initially described as a non-canonical RNA-dependent RNA polymerase and produced as part of the pp1a protein whether or not frameshifting occurs [32]. In contrast, the canonical RdRP Nsp12 is produced as part of the pp1ab protein only if frameshifting occurs. This, in conjunction with our data suggests that the nsp8 polymerse might direct sgRNA production and that the RdRP encoded in nsp12 is predominantly used for gRNA production. It has been hypothesized that coronavirus RNA synthesis involves structurally and functionally separable RNA synthesising complexes [33]. The nsp7+nsp8 complex from three different coronaviruses have recently been shown to have primer-independent RNA polymerase activity [33,34] in contrast to the nsp12 RdRP which is primer dependent [35]. Currently the details regarding coronavirus RNA synthesis are very limited and do not explain how the polymerases distinguish between templates or prime synthesis. te Velthuis and colleagues suggested that the separate RdRPs may influence plus and minus strand synthesis [33] but our results indicate that the division might be between genomic and subgenomic RNA synthesis. The RT-PCR for detection of gRNA and sgRNA in our study generated a cDNA intermediate using random hexamers and because of this the analyses performed here do not distinguish between the production of positive- and negative-strands nor between mRNA and sgRNA. Thus, we do not know if the regulatory sequences or proteins regulating genomic RNA production act upon the positive- or negative-strand replication intermediates or transcription intermediates.

A slight difference in frameshifting efficiency, such as that between the L2-UCC and S3D mutants, would not be expected to have such significant effects on viral RNA levels. Similar to previously described viruses containing mutations in the slippery site of the frameshift signal [7], here we show that mutations to the SARS-CoV frameshift stimulating mRNA pseudoknot can also affect the production of viral genomic RNA. In both instances, the abundance of viral genomic RNA was reduced to 10-fold lower levels than the subgenomic RNA and several orders of magnitude below that of the wild-type virus. It is not clear if this result is due to a change in the abundance of gRNA available to be transcribed to sgRNA or if it is attributable to other factors, such as interactions between the gRNA and other proteins or RNA elements as discussed above. It is also possible that the synonymous changes altered the rate of translation and/or the folding of the RdRP [36] which, in turn, could have affected production of genomic and/or subgenomic RNA. Regardless of the mechanism, our observations provide a backdrop against which new questions about coronvirus replication and transcription may be explored.
